# Radiocaesium in *Cortinarius* spp. mushrooms in the regions of the Reggio Emilia in Italy and Pomerania in Poland

**DOI:** 10.1007/s11356-016-7541-0

**Published:** 2016-09-06

**Authors:** Tamara Zalewska, Luigi Cocchi, Jerzy Falandysz

**Affiliations:** 1 Institute of Meteorology and Water Management–National Research Institute, Maritime Branch, Waszyngtona 42, 81-342 Gdynia, Poland; 2 Gruppo Micologico e Naturalistico R, FranchiVia D, Piani, 6, I-42100 Reggio Emilia, Italy; 3 Comitato Scientifico Nazionale dell’ Associazione Micologica Bresadola, Via A, Volta, 46, I-38100 Trento, Italy; 4 Laboratory of Environmental Chemistry & Ecotoxicology, Gdańsk University, 63 Witta Stwosza Street, PL 80-308 Gdańsk, Poland

**Keywords:** Europe, Edible fungi, Macromycetes, Radioactivity, Radiocaesium

## Abstract

Activity concentrations of ^134^Cs and ^137^Cs have been determined in 23 species of mushrooms of the genus *Cortinarius* (59 individual samples) collected from the Reggio Emilia in Italy 1992–1999 and in 4 species (16 composite samples and 413 individuals) from the Pomerania region in Poland from 1996 to 2015. Across all the *Cortinarius* species from the Reggio Emilia, the activity concentrations were relatively high in *Cortinarius alboviolaceus*, *Cortinarius duracinus*, *Cortinarius orellanus*, *Cortinarius rapaceus*, and *Cortinarius subannulatus*, in which ^137^Cs was at 10,000 ~ 100,000 Bq kg^−1^ dry biomass (db) in 1994. Smaller activity concentrations were found in *Cortinarius bivelus*, *Cortinarius bulliardii*, *Cortinarius cotoneus*, *Cortinarius largus*, *Cortinarius lividoviolaceus*, *Cortinarius purpureus*, *Cortinarius rufo-olivaceus*, *Cortinarius torvus*, and *Cortinarius venetus* with levels at 1000 ~ 6000 Bq kg^−1^ db from 1992 to 1994, and further in *Cortinarius anserinus*, *Cortinarius auroturbinatus*, *C*. *largus*, *Cortinarius praestans*, *Cortinarius purpurascens*, *Cortinarius scaurus*, *Cortinarius sebaceous*, *Cortinarius talus*, and *Cortinarius variecolor* with activity concentrations at 100 ~ 600 Bq kg^−1^ db in 1994. All the data were calculated for dehydrated fungal material corrected back to the exact date samples of collection. The greatest activity concentrations of ^137^Cs both in Italy (1992–1999) and Poland (1996–2010) were found in the popular *Cortinarius caperatus*, confirming its very high capacity of radiocaesium accumulation. Besides ^137^Cs, the isotope ^134^Cs was detected in some species from the Reggio Emilia. An average calculated ratio of activities of ^134^Cs to ^137^Cs referenced to 1986 was equal to 0.38 in mushrooms from the Reggio Emilia, and this value slightly differ from that specific for Chernobyl fallout, which was 0.54. It was calculated that ^137^Cs originating from Chernobyl accident constituted about 68 % of the total activity concentration of the isotope in Reggio Emilia in 1986, while as much as 32 % of ^137^Cs in mushrooms were from the global fallout from nuclear bomb testing.

## Introduction

The fallout from the atmospheric nuclear explosions of the twentieth century caused a global scale pollution with partly long-lived radionuclides (Bem et al. 1990; Grueter 1964; Saniewski et al. 2016). Further, two major scale nuclear power plant (NPP) accidents in Chernobyl (Ukraine, 1986) and Fukushima Daiichi (Japan, 2011) released radionuclides of various half-lives (Steinhauser 2014). The releases from the Chernobyl NPP exceeded the releases from Fukushima by one order of magnitude and caused more severe radiation related health effects (Steinhauser et al. 2014). The Chernobyl accident caused severe pollution of soils with caesium radioisotopes ^134^Cs and ^137^Cs in the vicinity of the nuclear facility and more or less in the neighboring regions in Europe (De Cort et al. 1998). The influence of the Fukushima accident on radioactivity levels in Europe was regarded as negligible (Steinhauser et al. 2013).

Mushrooms growing in the wild are specifically prone to contamination with several metallic elements (Falandysz and Borovička 2013). The nuclear weapon tests and Chernobyl accident caused a long-lasting accumulation of the radionuclides with long half-lives in soils and mushrooms. An especially high accumulation was observed for ^137^Cs (Bakken and Olsen 1990; Baldini and Nyatemu 1990; Bulko et al. 2014; Falandysz et al. 2015a; García et al. 2015; Grodzinskaya et al. 2003, 2013; Mietelski et al. 2010). Mushrooms absorb both natural and artificial radionuclides originating from fallout and from soil substrata, and ^137^Cs is often a major contaminant but because of species-specific accumulation some other radionuclides can also highly matter for some mushrooms and regions (Steinhauser et al. 2014; Kirchner et al. 1998).

Radiocaesium isotopes (^134^Cs and ^137^Cs) are rapidly accumulated by mushrooms from the soil contaminated with deposited fallout. This is because of a high similarity of ^134^Cs and ^137^Cs to stable Cs (^133^Cs)—a natural metallic trace element in mushrooms on one side and because of the very efficient mycelial network on the other side. There is also some chemical and physical analogy between the isotopes of Cs and the isotopes of potassium (K) which is the most abundant metallic macro element present in mushrooms. Potassium concentration was found to be in the range from 34,000 to 52,000 mg kg^−1^ dry biomass (db; median values) in caps of edible Brown Birch Scaber Stalk *Leccinum scabrum* (Falandysz et al. 2007a).

The rate or efficiency of ^134^Cs and ^137^Cs accumulation by mushrooms can be related to status of the stable Cs in their flesh, which seems to be species specific. For example, *Macrolepiota procera* (Scop.) Singer is poor in total Cs with a range in caps from 0.015 to 0.043 mg kg^−1^ db; *Amanita muscaria* **(**L.) Lam., with from 0.063 to 0.83 mg kg^−1^ db; *L. scabrum* with from 2.7 to 7.2 mg kg^−1^ db and *Sarcodon imbricatus* (L.) P. Karst., with 28 ± 9 mg kg^−1^ db (Kotłów et al. 2015; Falandysz et al. 2007a, b, 2008). This implies that differences in the efficiency of absorption of ^137^Cs by fungi and accumulation in fruiting bodies follow their stable Cs status, while surplus of “easily” available ^137^Cs in soil solution can also matter for high accumulation. In 2000, the authors Yoshida et al. reported on a good correlation between ^137^Cs and stable ^133^Cs contained in several species of mushrooms from Finland, Germany, and Japan (Yoshida et al. 2000).

Gypsy *Cortinarius caperatus* (Pers.) Fr (earlier called *Rozites caperatus* (Pers.) P. Karst.,) is one of many species of the genus *Cortinarius* (Index Fungorum 2015). This edible and tasty mushroom is popular in many regions of Europe (Falandysz 2014). Shortly after the collapse of the Chernobyl nuclear power plant, *C. caperatus* collected in Europe showed high levels of radioactivity because of accumulated radiocesium (Byrne 1998; Eckl et al. 1985; Haselwandter et al. 1988; Strandberg 1994, 2004).

Both, Italy and Poland were affected by radionuclides released from the Chernobyl accident but there is a variation of the amount of deposition of radiocaesium over Europe (De Cort et al. 1998). This study aimed to update and document information on radioactivity from radiocaesium accumulated in mushrooms of some *Cortinarius* species and especially in *C. caperatus* collected in the Reggio Emilia in Italy and Pomerania in Poland. Italy and Poland are among the European countries where foraging of mushrooms is a traditional activity.

## Materials and methods

The fruitbodies of the genus *Cortinarius* such as *Cortinarius alboviolaceus* (Pers.) Fr.; *Cortinarius anserinus* (Velen.) Rob. Henry; *Cortinarius auroturbinatus* (Secr.) J.F. Lange; *Cortinarius bivelus* (Fr.) Fr.; *Cortinarius bulliardii* (Pers.) Fr*.*; *Cortinarius cotoneus* Fr.; *Cortinarius duracinus* Fr., current name *Cortinarius rigens* (Pers.) Fr.; *Cortinarius herpeticus* for which current name is *Cortinarius scaurus* (Fr.) Fr.; *Cortinarius largus* Fr.; *Cortinarius lividoviolaceus* (Rob. Henry ex M.M. Moser) M.M. Moser; *Cortinarius melliolens* Jul. Schäff., with current name *Cortinarius talus* Fr.; *Cortinarius orellanus* Fr., *Cortinarius phoeniceus* with current name *Cortinarius purpureus* (Bull.) Bidaud, Moënne-Locc. & Reumaux; *Cortinarius praestans* (Cordier) Gillet; *Cortinarius purpurascens* Fr.; *Cortinarius rapaceus* Fr.; *Cortinarius rufo-olivaceus* (Pers.) Fr.; *Cortinarius sebaceus* Fr.; *Cortinarius subannulatus* Jul. Schäff. & M.M. Moser; *Cortinarius torvus* (Fr.) Fr.; *Cortinarius variecolor* (Pers.) Fr., and *Cortinarius venetus* (Fr.) Fr. were collected from the Reggio Emilia in Italy in 1992–1994, and *Cortinarius caperatus* (Pers.) Fr (earlier called *R. caperatus* (Pers.) P. Karst) in 1992–1995 and 1999 (coordinates: 44° 42′ N 10° 38′ E) (Table 1). The fruitbodies of *C. caperatus* were also collected from Pomerania in Poland in 1996–2010 (coordinates: 54° 29′ N 18° 15′ E). Additional samples of *Cortinarius* species such as *Cortinarius agathosmus* Brandrud, H. Lindstr. & Melot, *Cortinarius olivaceofuscus* Kühner, *Cortinarius trivialis* J.E. Lange, and *Cortinarius violaceus* (L.) Gray were gathered in 2013 and in 2015 in region of the village of Pomlewo (Pomerania; coordinates: 54° 13′ 07″ N 18° 21′ 28″ E) (Table 1).

**Table 1:** Activity concentration of ^137^Cs in *Cortinarius* spp. from the Reggio Emilia in Italy and the region of the Pomerania land in Poland (Bq kg^−1^ dry biomass)

Localization, species, date or year and sample size (*n* = number of fruiting bodies)	Whole fruiting bodies		
	Caps		Stipes
Italy; *C. alboviolaceus*, 1994–09-04/10–04 *n* = 2^#^		12,000 ± 60 (12,000–12,000)	
Italy; *C*. *anserinus*, 1994–09-19 *n* = 1^#^		120 ± 10	
Italy; *C*. *auroturbinatus*, 1994–11-25 *n* = 1^#^		570 ± 20	
Italy; *C*. *bivelus*, 1994–09-19 *n* = 1^#^		3200 ± 28	
Italy; *C*. *bulliardii*, 1993–11-08 *n* = 1^#^		2600 ± 30	
Italy; *C. caperatus*, 1992–10-01/12/02/12 *n* = 6^#^		33,000 ± 35,000 (6300–100,000) 21,000^*^	
Italy; *C. caperatus*, 1993–08-15/10–02/15 *n* = 3^#^		15,000 ± 8000 (5800–21,000) 18,000	
Italy; *C. caperatus*, 1994–08-10/07/28/09–26/10–01/02 *n* = 6^#^		9900 ± 5360 (3300–18,800) 9900	
Italy; *C. caperatus*, 1995–09-02/09/17 *n* = 16^#^		13,000 ± 3900 (5800–16,000) 14,000	
Italy; *C. caperatus*, 1999–07-29/10–24 *n* = 2^#^		17,000 (15,000–19,000)	
Italy; *C*. *cotoneus*, 1994–09-19/10–12 n = 3^#^		3100 ± 2700 (340–5800) 3200	
Italy; *C*. *duracinus*, 1994–11-25 *n* = 1^#^		12,000 ± 100	
Italy; *C*. *herpeticus* (*C. scaurus*), 1994–09-19 *n* = 1^#^		290 ± 20	
Italy; *C*. *largus*, 1992–10-25 *n* = 1^#^		220 ± 13	
Italy; *C*. *largus*, 1994–10-16 *n* = 1^#^		1100 ± 20	
Italy; *C*. *lividoviolaceus*, 1994–09-19 *n* = 1^#^		1700 ± 25	
Italy; *C*. *melliolens* (*C. talus*), 1994–10-14 n = 1^#^		180 ± 10	
Italy; *C*. *orellanus*, 1994–10-25 *n* = 1^#^		26,000 ± 180	
Italy; *C. phoeniceus* (*C. purpureus*), 1994–09-19 n = 1^#^		2700 ± 28	
Italy; *C. praestans*, 1993–09-24 n = 1^#^		130 ± 9	
Italy; *C. purpurascens*, 1994–09-19 n = 1^#^		220 ± 12	
Italy; *C*. *rapaceus*, 1994–10-15 *n* = 1^#^		31,000 ± 200	
Italy; *C*. *rufo-olivaceus*, 1994–09-19 *n* = 1^#^		2400 ± 23	
Italy; *C*. *sebaceus*, 1994–09-19 *n* = 1^#^		380 ± 19	
Italy; *C*. *subannulatus*, 1994–10-01 *n* = 1^#^		18,000 ± 130	
Italy; *C. torvus*, 1994–10-08 *n* = 1^#^		5600 ± 50	
Italy; *C*. *variecolor*, 1994–09-19 *n* = 1^#^		320 ± 17	
Italy; *C*. *venetus*, 1994–10-14 *n* = 1^#^		5600 ± 44	
Poland; *C. caerulescens*, Pomlewo, 2013 *n* = 3^##^		18 ± 4^**^	
Poland; *C. caerulescens*, Pomlewo, 2015 *n* = 25^##^	5.8 ± 1.0		5.1 ± 1.0
Poland; *C. caperatus*, TLP, 1996 *n* = 20^##^	13,000 ± 110		4200 ± 45
Poland; *C. caperatus*, WLP, 1998 *n* = 15^##^	16,000 ± 180		5300 ± 65
Poland; *C. caperatus*, Darżlubska Wilderness, 2001 *n* = 15^##^	6500 ± 60		2000 ± 19
Poland; *C. caperatus*, Przymuszewo, 2002 *n* = 16^##^	12,000 ± 94		5400 ± 43
Poland; *C. caperatus*, Darżlubska Wilderness, 2003 *n* = 15^##^	5100 ± 43		2100 ± 19
Poland; *C. caperatus*, Słupsk county, Kępice, 2003 *n* = 31^##^	5200 ± 49		2000 ± 19
Poland; *C. caperatus*, Sulęczyno, 2006 *n* = 70^##^	5200 ± 50		3500 ± 25
Poland; *C. caperatus*, Strzebielino, 2006 *n* = 16^##^	4600 ± 42		1800 ± 23
Poland; *C. caperatus*, Tuchola Pinewoods, Lubichowo, 2007 *n* = 53^##^	7300 ± 58		2900 ± 24
Poland; *C. caperatus*, Gołubie, 2008 *n* = 15^##^	6500 ± 47		3000 ± 22
Poland; *C. caperatus*, Tuchola Pinewoods, Lubichowo, 2010 *n* = 16^##^	5600 ± 58		1900 ± 24
Poland; *C. olivaceofuscus* Pomlewo, 2013 *n* = 14^##^		10 ± 2	
Poland; *C. olivaceofuscus* Pomlewo, 2015 *n* = 54^##^	9.2 ± 2.0		6.9 ± 1.6
Poland; *C. trivialis*, Pomlewo, 2015 *n* = 35^##^		93 ± 2	

Notes: ^#^Number of individual samples; ^##^Number of individuals in a pool; TLP (Trójmiejski Landscape Park); WLP (Wdzydze Landscape Park);

^*^Mean ± S.D. (range) and median value; ^**^Measurement uncertainty

The individual cap, stipe, or a whole fruiting bodies samples were cleaned from plant and soil debris using a soft brush and a disposable plastic knife, dried, and ground (Cocchi et al. 2006; Falandysz 2014). In the case of 23 species of mushrooms from the Reggio Emilia in Italy, a single whole fruiting body was examined for each species (in total 59 individual samples), and from the Pomerania region, 16 composite samples were examined, which were prepared using from 3 to 70 individuals per location (Table 1). The activity concentrations of ^134^Cs and ^137^Cs in samples collected in Italy and Poland were determined using a validated method of gamma spectrometry with coaxial high purity germanium (HPGe) detectors.

Details on the gamma spectrometer calibration, detector parameters, and quality assurance of the measurements and instrument used in Italy [HPGe high purity detectors (P-type HPGe and HPGe-N-EG G ORTEC, relative efficiencies between 25 and 80 % and resolution of approximately 2.0 keV (FWHM) at 1:33 MeV energy), shielded by lead walls (thickness about 10 cm), calibrated with multirange source QCY 48 (energy range between 59 keV and 1.836 MeV) and equipped with spectra processing program (GammaVision 7:02-ORTEC)] were provided in a paper by Consiglio et al. (1990). For instrument used in Poland, the germanium detector relative efficiency was 18 % and a resolution of 1.9 keV at 1.332 MeV (with associated electronics). The equipment was calibrated using a multi-isotope standard and the method was fully validated. The reference solution, “Standard solution of gamma emitting isotopes, code BW/Z-62/27/07” produced at the IBJ-Świerk near/Otwock in Poland, was used for preparing reference samples for equipment calibration. The same geometry of cylindrical dishes with 40 mm diameter (as applied for environmental samples) was used for reference samples during equipment calibration. The laboratory involved was subject to routine checks to ensure high standards of analytical quality and analytical control and participated successfully in the inter-comparison exercises organized by IAEA-MEL Monaco (IAEA-414, Irish and North Sea Fish) to confirm the reliability and accuracy of the method (Falandysz et al. 2015a, b; Wang et al. 2015; Zalewska and Saniewski 2011). Repeated analysis gave values of ^137^Cs—5.06 ± 0.64 Bq kg^−1^ db, ^4^°K—474.5 ± 19.3 Bq kg^−1^ db, while the estimated target value was equal to 5.18 ± 0.10 Bq kg^−1^ db for ^137^Cs and 481 ± 16 Bq kg^−1^ db for ^4^°K. In the gamma spectrometry measurements, the limit of quantification was calculated using GENIE 2000 as a Minimum Detectable Activity (MDA) defined by Curie (1968). Data were calculated for dehydrated fungal material (at 105 °C) and corrected back to the exact date of sample collection.

## Results and discussion

Table 1 presents all the mushroom names, site locality, year of collection, number of fruitbodies, and measurement data. The fruitbodies collected in the Reggio Emilia in the period from 1992 to 1994 showed relatively high activity concentrations of ^137^Cs (half-life T_1/2_ 30.05 years) at a range from 120 Bq kg^−1^ db in *C*. *anserinus* to 31,000 Bq kg^−1^ db in *C. rapaceus*, with both collected in 1994 (Table 1). If recalculated to fresh weight (assuming a moisture content of fruiting bodies at 90 %), the values would be 12 Bq kg^−1^ for *C*. *anserinus* and 3100 Bq kg^−1^ for *C. rapaceus*.

In some *Cortinarius* species, in Reggio Emilia, ^134^Cs (half-life T_1/2_ 2.06 years) was detected, but activity concentrations were visibly lower. They ranged from 8 Bq kg^−1^ db in *C. cotoneus* to 987 Bq kg^−1^ db in *C. rapaceus*, and the latter showed also the greatest ^137^Cs activity concentration. It has been shown that there is a relationship between the activity concentration of both isotopes in mushrooms (fruiting bodies). The ratio of activity concentrations of ^134^Cs to concentrations of ^137^Cs referenced to 1986 varied within a narrow range from 0.29 to 0.46. The averaged value was 0.38. It should be noted that this value only slightly differs from that specific for the Chernobyl fallout, which was 0.54 (Aarkrog et al. 1988; Strandberg 2004). Taking into account the value specific for the Chernobyl fallout and activity concentrations of ^134^Cs referenced to 1986, it is possible to calculate activity concentrations of ^137^Cs originating from the Chernobyl accident. It was calculated that this ^137^Cs constituted about 63 % of the total activity concentration of the isotope present in 1986 in fruiting bodies of examined mushrooms (the range is from 53 to 85 %). This means that as much as 32 % of ^137^Cs in mushrooms were from the global fallout from nuclear bomb testing.

The species *C*. *anserinus*, *C*. *auroturbinatus*, *C. scaurus* (*herpeticus*), *C*. *largus* (one sample), *C*. (*talus*) *melliolens*, *C. praestans*, *C. purpurascens*, *C*. *sebaceus*, and *C*. *variecolor* did not show ^134^Cs at a level > 0.1 Bq kg^−1^ db, while activity levels of ^137^Cs were <300 Bq kg^−1^ db (Table 1). *C. caperatus* collected in Poland did not show activity concentrations from ^134^Cs at the time of analysis (spring 2012).

Across all the *Cortinarius* sporocarps examined from Reggio Emilia, the activity concentrations were relatively high in *C. alboviolaceus*, *C. caperatus*, C. *duracinus*, *C*. *rapaceus*, and *C*. *subannulatus* in which ^137^Cs was at 10,000 ~ 100,000 Bq kg^−1^ db. Smaller activity concentrations were found in *C. bivelus*, *C. bulliardii*, *C. cotoneus*, *C. largus*, *C. lividoviolaceus*, *C. phoeniceus* (*C. purpureus*), *C. rufo-olivaceus*, *C. torvus*, and *C. venetus* with levels at 1000 ~ 6000 Bq kg^−1^ db, and further in *C*. *anserinus*, *C*. *auroturbinatus*, C. *herpeticus* (*C. scaurus*), *C*. *herpeticus* (*C. scaurus*), *C*. *largus*, *C*. *melliolens* (*C. talus*), *C. praestans*, *C. purpurascens*, *C*. *sebaceus*, and *C*. *variecolor* with activity concentrations at 100 ~ 600 Bq kg^−1^ db (Table 1).

*Cortinarius caperatus* and also *Cortinarius armillatus* (Fr.) Fr., highly accumulated pre- and post-Chernobyl ^137^Cs. ^[25–26]^ *Cortinarius* fruitbodies of *C. caperatus*, *C. armillatus*, and *Cortinarius traganus* (Fr.) Fr., were very efficient accumulators of ^134/137^Cs (median value of ^137^Cs in individuals from Slovenia was respectively at 22,000, 44,000, and 12,000 Bq kg^−1^ db), while *C. praestans* was a weak accumulator, as indicated by our results and a study by Byrne (1998). Our results for *C. caperatus* as efficient accumulators of ^137^Cs agreed with data by Strandberg (1994 and 2004), while that for *C. armillatus* this is new record. The largest amount of data on ^137^Cs in this study concerns *C. caperatus*, both from the Reggio Emilia in Italy and Pomerania in Poland (Table 1). This species accumulates ^137^Cs much more efficiently in caps than in stipes and the range of the values of ^137^Cs cap to stipe activity concentration quotient (Q_C/S_) for the fruitbodies collected in Pomerania was from 1.4 to 3.1.

The highest activity concentration of ^137^Cs (16,000 Bq kg^−1^ db) in caps of *C. caperatus* from northern Poland was identified in a sample gathered in 1998. High activity concentrations were also found in individuals collected in 1996 (13,000 Bq kg^−1^ db) and in 2002 (12,000 Bq kg^−1^ db). The activity concentrations of ^137^Cs in most samples collected in the period from 2001 until 2010 remained in the range from 3800 to 9000 Bq kg^−1^ db. Regarding the northern Polish region, the lowest ^137^Cs activity concentrations were determined for other species of the genus *Cortinarius* that were collected in 2013 and 2015. In 2013, ^137^Cs in fruiting bodies of *C. olivaceofuscus* was at 11 Bq kg^−1^ db and in *C. caerulescens* at 18 Bq kg^−1^ db. In 2015, the activity concentration in *C. trivialis* was at 93 Bq kg^−1^ db, while the caps and stipes of *C. caerulescens* and *C. trivialis* were at a level of a few Bq kg^−1^ db (Table 1).

There were some similarities in the ^137^Cs activity concentrations in *C. caperatus* collected in the Reggio Emilia in 1995 and 1999 (at 14,000 and 17,000 Bq kg^−1^ db or 1400 and 1700 Bq kg^−1^ fresh weight base, respectively) and fruitbodies collected in 1996–1998 in Pomerania (in caps at 13,000–16,000 Bq kg^−1^ db or 1300–1600 Bq kg^−1^ fresh weight base) (Table 1). The fruitbodies of *C. caperatus* sampled in Denmark in 1994 contained ^137^Cs at 13,000 Bq kg^−1^ db, while individuals from a montane region of Norway sampled in 1994 contained ^137^Cs at 7700 Bq kg^−1^ db (median value) (Strandberg 1994 and 2004).

Recently, coral fungi of the genus *Clavaria* were reported as efficient accumulators of radiocaesium and samples collected in Japan after the Fukushima nuclear accident showed radiocaesium at 28,000 Bq kg^−1^ fresh weight base (Merz et al. 2015).

## Conclusion

The greatest activity concentrations of ^137^Cs in mushrooms of the genus *Cortinarius* collected both in the Reggio Emilia in Italy and in the region of northern Poland were found in *C. caperatus*, which is one of the most popular species from this genus, and it’s very high capacity for radiocaesium accumulation was confirmed. Besides ^137^Cs, the isotope ^134^Cs was detected in some species from Italy. An average calculated ratio of activities of ^134^Cs to ^137^Cs referenced to 1986 was equal to 0.38 in mushrooms from the Reggio Emilia, and this value slightly differs from that specific for the Chernobyl fallout, which was 0.54. It was calculated that ^137^Cs originating from the Chernobyl accident constituted about 68 % of the total activity concentration of the isotope in Reggio Emilia in 1986, while as much as 32 % of ^137^Cs in mushrooms resulted from the global fallout from nuclear bomb testing.

## References

[CR1] Aarkrog A. Bøtter-Jensen L, Qing Jiang C, Dahlgaard H, Hansen H, Holm E, Lauridsen B, Nielsen SP, Søegaard-Hansen J (1988) Environmental Radioactivity in Denmark in 1986. Risø Report 1988. RISØ-R-549

[CR2] Bakken LR, Olsen RA (1990). Accumulation of radiocaesium in fungi. Can. J Microbiol.

[CR3] Baldini E, Nyatemu K (1990). Tubertini, O. ^137^Cs/^134^Cs anomalous ratios in organic soils and mushrooms affected by Chernobyl pollution. Radiochim Acta.

[CR4] Bem H, Lasota W, Kuśmierek E, Witusik M (1990). Accumulation of 137Cs by mushrooms from Rogożno area of Poland over the period 1984-1988. J Radioanal Nucl Chem Lett.

[CR5] Bulko NI, Shabaleva M, Kozlov AK, Tolkacheva NV, Mashkov LA (2014). The ^137^Cs accumulation by forest-derived products in the Gomel region. J Environ Radioact.

[CR6] Byrne AR (1998). Radioactivity in fungi in Slovenia, Yugoslavia, following the Chernobyl accident. J Environ Radioac.

[CR7] Cocchi L, Vescovi L, Petrini L, Petrini O (2006). Heavy metals in edible mushrooms in Italy. Food Chem.

[CR8] Consiglio G, Gattavecchia E, Tonelli D, Cocchi L (1990). La radioattività nei funghi: attualità del problema ed opportunità di un approfondimento. Riv Micol.

[CR9] Curie LA (1968). Limits for qualitative detection and quantitative determination. Application to radiochemistry. Anal Chem.

[CR10] De Cort M, Dubois G, Fridman SD, Germenchuk MG, Izrael YA, Janssens A, Jones AR, Kelly GN, Kvasnikova EV, Matvenko II, Nazarov IM, Pokumeiko YM, Sitak VA, Stukin ED, Tabuchny LY, Tsaturov YS, Avdyushin SI (1998). Atlas of caesium deposition on Europe after the Chernobyl accident. Luxembourg: Office for Official Publications of the European. Communities.

[CR11] Eckl P, Hofmann W, Turk R (1985). Uptake of natural and man-made radionuclides by lichens and mushrooms. Radiat Environ Biophys.

[CR12] Falandysz J (2014). Distribution of mercury in gypsy *Cortinarius caperatus* mushrooms from several populations: an efficient accumulator species and estimated intake of element. Ecotox Environ Saf.

[CR13] Falandysz J, Borovička J (2013). Macro and trace mineral constituents and radionuclides in mushrooms—health benefits and risks. Appl Microbiol Biotechnol.

[CR14] Falandysz J, Kunito T, Kubota R, Bielawski L, Mazur A, Falandysz JJ, Tanabe S (2007). Selected elements in Brown birch Scaber stalk *Leccinum scabrum*. J Environ Sci Health Part A.

[CR15] Falandysz J, Kunito T, Kubota R, Lipka K, Mazur A, Falandysz JJ, Tanabe S (2007). Selected elements in fly agaric *Amanita muscaria*. J Environ Sci Health Part A.

[CR16] Falandysz J, Kunito T, Kubota R, Gucia M, Mazur A, Falandysz JJ, Tanabe S (2008). Some mineral constituents of parasol mushroom *Macrolepiota procera*. J Environ Sci Health Part B.

[CR17] Falandysz J, Zalewska T, Krasińska G, Apanel A, Wang Y, Pankavec S (2015). Evaluation of the radioactive contamination in fungi genus *Boletus* in the region of Europe and Yunnan Province in China. Appl Microbiol Biotechnol.

[CR18] Falandysz J, Zhang J, Zalewska T, Apanel A, Wang Y, Wiejak A (2015). Distribution and possible dietary intake of radioactive ^137^Cs, ^4^°K and ^226^Ra with the pantropical mushroom *Macrocybe gigantea* in SW China. J Environ Sci Health Part A.

[CR19] Index Fungorum (2015) http://www.indexfungorum.org/names/Names.asp (accessed on November 12, 2015)

[CR20] García MA, Alonso J, Melgar MJ (2015). Radiocaesium activity concentrations in macrofungi from Galicia (NW Spain): influence of environmental and genetic factors. Ecotox Environ Saf.

[CR21] Grodzinskaya AA, Berreck M, Haselwandter K, Wasser SP (2003). Radiocesium contamination of wild-growing medicinal mushrooms in Ukraine. Int J Med Mush.

[CR22] Grodzinskaya AA, Syrchin SA, Kuchma ND, Wasser SP (2013) Macromycetes accumulative activity in radionuclide contamination conditions of the Ukraine territory. Part 6. - P.217–260, 368–373. In: Mycobiota of Ukrainian Polesie: Consequences of the Chernobyl disaster. Kiev: Naukova dumka (In Russian)

[CR23] Grueter H (1964). Eine selektive Anreicherung des Spaltprodukts ^137^Cs in Pilzen. Naturwissenachaften.

[CR24] Haselwandter K, Berreck M, Brunner P (1988). Fungi as bioindicators of radiocaesium contamination: pre- and post-Chernobyl activities. Trans Br Mycol Soc.

[CR25] Kirchner G, Daillant O (1998). Accumulation of 210Pb, 226Ra and radioactive cesium by fungi. Sci Total Environ.

[CR26] Kotłów J, Chudzińska M, Barałkiewicz D, Falandysz J (2015) Badania nad składem i współzależnościami występowania wybranych pierwiastków śladowych w grzybie *Sarcodon imbricatus*. XI Międzynarodowa Konferencja Naukowo-Techniczna. „Obieg pierwiastków w przyrodzie – bioakumulacja – toksyczność – przeciwdziałanie”. 10 września 2015 r. Warszawa. IOŚ – PIB; Instytut Ochrony Środowiska – Państwowy Instytut Badawczy. Streszczenia posterów, 64

[CR27] Merz S, Shozugawa K, Steinhauser G (2015). Analysis of Japanese radionuclide monitoring data of food before and after the Fukushima nuclear accident. Environ Sci Technol.

[CR28] Mietelski JW, Dubchak S, Błażej S, Anielska T, Turnau K (2010). ^137^Cs and ^4^°K in fruiting bodies of different fungal species collected in a single forest in southern Poland. J Environ Radioact.

[CR29] Saniewski M, Zalewska T, Krasińska G, Szylke N, Wang Y, Falandysz J (2016). ^90^Sr in king bolete *Boletus edulis* and certain other mushrooms consumed in Europe and China. Sci Total Environ.

[CR30] Steinhauser G (2014). Fukushima’s forgotten radionuclides: a review of the understudied radioactive emissions. Environ Sci Technol.

[CR31] Steinhauser G, Merz S, Hainz D, Sterba JH (2013). Artificial radioactivity in environmental media (air, rainwater, soil, vegetation) in Austria after the Fukushima nuclear accident. Environ Sci Pollut Res.

[CR32] Steinhauser G, Brandl A, Johnson TE (2014). Comparison of the Chernobyl and Fukushima nuclear accidents: a review of the environmental impacts. Sci Total Environ.

[CR33] Strandberg M (1994). Radiocesium in a Danish pine forest ecosystem. Sci Total Environ.

[CR34] Strandberg M (2004). Long-term trends in the uptake of radiocesium in *Rozites caperatus*. Sci Total Environ.

[CR35] Wang Y, Zalewska T, Apanel A, Zhang J, Wiejak A, Falandysz J (2015). ^137^Cs, ^134^Cs and natural ^4^°K in sclerotia of *Wolfiporia extensa* fungus collected across of the Yunnan land in China. J Environ Sci Health Part B.

[CR36] Yoshida S, Muramatsu Y, Steiner M, Belli M, Pasquale A, Rafferty B, Rühm W, Rantavaara A, Linkov I, Dvornik A, Zhuchenko T (2000) *In* Relationship between radiocesium and stable cesium in plants and mushrooms collected from forest ecosystems with different contamination levels. Proceedings of the 10th International Congress of the International Radiation Protection Association, May, 2000; Hiroshima P-11–244

[CR37] Zalewska T, Saniewski M (2011). Bioaccumulation of gamma emitting radionuclides in red algae from the Baltic Sea under laboratory conditions. Oceanologia.

